# 2-Nitro-*p*-phenyl­ene bis­(toluene­sulfonate)

**DOI:** 10.1107/S1600536808026573

**Published:** 2008-08-23

**Authors:** Xiujie Ji, Bowen Cheng, Jun Song, Chao Liu

**Affiliations:** a Tianjin Municipal Key Laboratory of Fiber Modification and Functional Fibers, Tianjin Polytechnic University, Tianjin 300160People’s Republic of China; b School of Materials Science and Engineering, Hebei University of Technology, Tianjin 300130, People’s Republic of China

## Abstract

In the mol­ecule of the title compound, C_20_H_17_NO_8_S_2_, the two toluene rings are oriented at a dihedral angle of 3.65 (4)°, while the nitrophenyl ring is oriented at dihedral angles of 44.39 (3) and 47.44 (3)° with respect to the toluene rings. An intra­molecular C—H⋯O hydrogen bond results in the formation of a five-membered ring, which adopts an envelope conformation. In the crystal structure, inter­molecular C—H⋯O hydrogen bonds link the mol­ecules. There is a π–π contact between the toluene rings [centroid–centroid distance = 4.035 (1) Å].

## Related literature

For related literature, see: Atkinson *et al.* (2005 ▶); Hu *et al.* (2001 ▶); Svensson *et al.* (1998 ▶); Trollsås *et al.* (1996 ▶). For bond-length data, see: Allen *et al.* (1987 ▶).
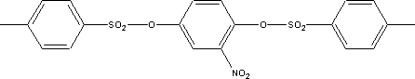


## Experimental

### 

#### Crystal data

C_20_H_17_NO_8_S_2_
*M* *_r_* = 463.49
Triclinic, 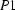

*a* = 7.926 (3) Å
*b* = 8.244 (3) Å
*c* = 8.709 (3) Å
α = 98.323 (6)°
β = 96.180 (6)°
γ = 103.915 (6)°
*V* = 540.5 (3) Å^3^
*Z* = 1
Mo *K*α radiation
μ = 0.29 mm^−1^
*T* = 294 (2) K
0.26 × 0.22 × 0.16 mm

#### Data collection

Bruker SMART CCD area-detector diffractometer
Absorption correction: multi-scan (*SADABS*; Sheldrick, 1996 ▶) *T*_min_ = 0.928, *T*_max_ = 0.955
2693 measured reflections
1918 independent reflections
1717 reflections with *I* > 2σ(*I*)
*R*_int_ = 0.018

#### Refinement

*R*[*F* ^2^ > 2σ(*F* ^2^)] = 0.045
*wR*(*F* ^2^) = 0.135
*S* = 1.05
1918 reflections
282 parameters
H-atom parameters constrained
Δρ_max_ = 0.38 e Å^−3^
Δρ_min_ = −0.29 e Å^−3^
Absolute structure: Flack (1983 ▶), 315 Friedel Pairs
Flack parameter: 0.21 (13)

### 

Data collection: *SMART* (Bruker, 1997 ▶); cell refinement: *SAINT* (Bruker, 1997 ▶); data reduction: *SAINT*; program(s) used to solve structure: *SHELXS97* (Sheldrick, 2008 ▶); program(s) used to refine structure: *SHELXL97* (Sheldrick, 2008 ▶); molecular graphics: *SHELXTL* (Sheldrick, 2008 ▶) and *PLATON* (Spek, 2003 ▶); software used to prepare material for publication: *SHELXTL*.

## Supplementary Material

Crystal structure: contains datablocks global, I. DOI: 10.1107/S1600536808026573/hk2500sup1.cif

Structure factors: contains datablocks I. DOI: 10.1107/S1600536808026573/hk2500Isup2.hkl

Additional supplementary materials:  crystallographic information; 3D view; checkCIF report

## Figures and Tables

**Table 1:** Hydrogen-bond geometry (Å, °)

*D*—H⋯*A*	*D*—H	H⋯*A*	*D*⋯*A*	*D*—H⋯*A*
C5—H5⋯O2^i^	0.93	2.49	3.405 (11)	168
C9—H9⋯O6^ii^	0.93	2.46	3.319 (9)	153
C10—H10⋯O8^i^	0.93	2.47	3.369 (10)	164
C11—H11⋯O1^iii^	0.93	2.50	3.352 (10)	153
C15—H15⋯O5	0.93	2.56	2.923 (10)	104
C18—H18⋯O5^iv^	0.93	2.49	3.409 (11)	168

Symmetry codes: (i) 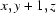; (ii) 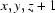; (iii) 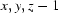; (iv) 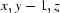.

**Fig. 1.**
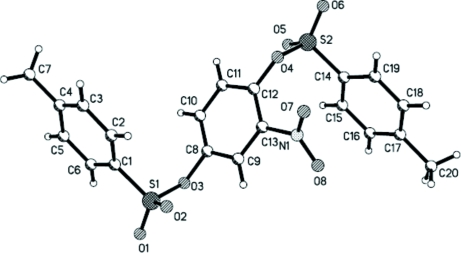
The molecular structure of the title molecule, with the atom-numbering scheme. Displacement ellipsoids are drawn at the 30% probability level.

**Fig. 2.**
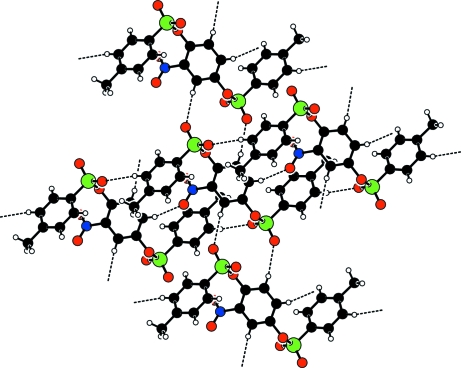
A packing diagram of the title compound. Hydrogen bonds are shown as dashed lines.

## supplementary crystallographic information

### Comment

Phenolic esters are useful intermediates in organic synthesis (Trollsås *et al.*, 1996; Svensson *et al.*, 1998; Atkinson *et al.*, 2005; Hu *et al.*, 2001). We have developed a new method for the syntheses of some phenolic esters. The title compound has been produced as a byproduct. We report herein its crystal structure.

In the molecule of the title compound (Fig. 1), the bond lengths (Allen *et al.*, 1987) and angles are within normal ranges. Rings A (C1—C6), B (C8—C13) and C (C14—C19) are, of course, planar, and the dihedral angles between them are A/B = 44.39 (3)°, A/C = 3.65 (4)° and B/C = 47.44 (3)°. The intramolecular C—H···O hydrogen bond (Table 1) results in the formation of a five-membered ring D (S2/O5/C14/C15/H15), adopting envelope conformation, with O5 atom displaced by 0.356 (3) Å from the plane of the other ring atoms.

In the crystal structure, intermolecular C—H···O hydrogen bonds (Table 1) link the molecules (Fig. 2), in which they may be effective in the stabilization of the structure. A π—π contact between A and C rings *Cg*1···*Cg*3^i^ [symmetry code: (i) 1 + *x*, 1 + *y*, 1 + *z*, where *Cg*1 and *Cg*3 are the centroids of the rings A and C, respectively] further stabilize the structure, with centroid-centroid distance of 4.035 (1) Å.

### Experimental

For the preparation of the title compound, 2-nitrohydroquinone (155 mg, 1.0 mmol) was dissolved in chloroform (30 ml). To this solution, 4-toluenesulfonyl chloride (191 mg, 1.0 mmol) and triethylamine (101 mg, 1.0 mmol) were added, the reaction was stirred at room temperature for 3 h. The reaction mixture was extracted with dichloromethane and dried with anhydrous sodium sulfate. After concentration, the residue was separated by flash column chromatography and purified by recrystallization from chloroform (yield; 72 mg, 31%, m.p. 415 K). Spectroscopic analysis: IR (KBr, ν, cm^-1^): 3089, 2922, 1596, 1539, 1390, 1201, 1183, 1089. Analysis required for C_20_H_17_NO_8_S_2_: C 51.83; H 3.70; N 3.02%. Found: C 50.85; H 3.65; N 3.19%.

### Refinement

H atoms were positioned geometrically, with C—H = 0.93 and 0.96 Å for aromatic and methyl H, respectively, and constrained to ride on their parent atoms with *U*_iso_(H) = *xU*_eq_(C), where *x* = 1.5 for methyl H and *x* = 1.2 for aromatic H atoms.

### Crystal data

**Table:**

C_20_H_17_NO_8_S_2_	*Z* = 1
*M**_r_* = 463.49	*F*_000_ = 240
Triclinic, *P*1	*D*_x_ = 1.424 Mg m^−^^3^
Hall symbol: P 1	Melting point: 415 K
*a* = 7.926 (3) Å	Mo *K*α radiation λ = 0.71073 Å
*b* = 8.244 (3) Å	Cell parameters from 1246 reflections
*c* = 8.709 (3) Å	θ = 2.6–26.9º
α = 98.323 (6)º	µ = 0.29 mm^−^^1^
β = 96.180 (6)º	*T* = 294 (2) K
γ = 103.915 (6)º	Block, colorless
*V* = 540.5 (3) Å^3^	0.26 × 0.22 × 0.16 mm

### Data collection

**Table:**

Bruker SMART CCD area-detector diffractometer	1918 independent reflections
Radiation source: fine-focus sealed tube	1717 reflections with *I* > 2σ(*I*)
Monochromator: graphite	*R*_int_ = 0.018
*T* = 294(2) K	θ_max_ = 25.0º
φ and ω scans	θ_min_ = 2.4º
Absorption correction: multi-scan(SADABS; Sheldrick, 1996)	*h* = −9→6
*T*_min_ = 0.928, *T*_max_ = 0.955	*k* = −7→9
2693 measured reflections	*l* = −10→8

### Refinement

**Table:**

Refinement on *F*^2^	Hydrogen site location: inferred from neighbouring sites
Least-squares matrix: full	H-atom parameters constrained
*R*[*F*^2^ > 2σ(*F*^2^)] = 0.045	*w* = 1/[σ^2^(*F*_o_^2^) + (0.0884*P*)^2^ + 0.0596*P*] where *P* = (*F*_o_^2^ + 2*F*_c_^2^)/3
*wR*(*F*^2^) = 0.135	(Δ/σ)_max_ = 0.007
*S* = 1.05	Δρ_max_ = 0.38 e Å^−^^3^
1918 reflections	Δρ_min_ = −0.29 e Å^−^^3^
282 parameters	Extinction correction: none
Primary atom site location: structure-invariant direct methods	Absolute structure: Flack (1983), with 315 Friedel Pairs
Secondary atom site location: difference Fourier map	Flack parameter: 0.21 (13)

### Special details

**Table:**

Geometry. All e.s.d.'s (except the e.s.d. in the dihedral angle between two l.s. planes) are estimated using the full covariance matrix. The cell e.s.d.'s are taken into account individually in the estimation of e.s.d.'s in distances, angles and torsion angles; correlations between e.s.d.'s in cell parameters are only used when they are defined by crystal symmetry. An approximate (isotropic) treatment of cell e.s.d.'s is used for estimating e.s.d.'s involving l.s. planes.
Refinement. Refinement of *F*^2^ against ALL reflections. The weighted *R*-factor *wR* and goodness of fit *S* are based on *F*^2^, conventional *R*-factors *R* are based on *F*, with *F* set to zero for negative *F*^2^. The threshold expression of *F*^2^ > σ(*F*^2^) is used only for calculating *R*-factors(gt) *etc*. and is not relevant to the choice of reflections for refinement. *R*-factors based on *F*^2^ are statistically about twice as large as those based on *F*, and *R*- factors based on ALL data will be even larger.

### Fractional atomic coordinates and isotropic or equivalent isotropic displacement parameters (Å^2^)

**Table:**

	*x*	*y*	*z*	*U*_iso_*/*U*_eq_	
S1	0.2533 (2)	0.96499 (17)	1.25707 (16)	0.0455 (6)	
S2	−0.0820 (2)	0.52158 (16)	0.40141 (16)	0.0461 (6)	
O1	0.1899 (10)	1.0093 (7)	1.3993 (6)	0.0661 (19)	
O2	0.3449 (8)	0.8397 (6)	1.2462 (6)	0.0602 (18)	
O3	0.0766 (7)	0.8988 (6)	1.1351 (6)	0.0461 (14)	
O4	0.0995 (7)	0.5877 (7)	0.5288 (6)	0.0477 (15)	
O5	−0.1792 (8)	0.6481 (6)	0.4198 (6)	0.0602 (17)	
O6	−0.0204 (8)	0.4733 (7)	0.2588 (6)	0.0578 (16)	
O7	0.2194 (9)	0.3656 (8)	0.7024 (7)	0.103 (2)	
O8	0.0174 (10)	0.3014 (7)	0.8493 (7)	0.104 (2)	
N1	0.1160 (10)	0.4007 (7)	0.7819 (7)	0.0713 (17)	
C1	0.3630 (12)	1.1486 (10)	1.1911 (10)	0.043 (2)	
C2	0.4931 (12)	1.1343 (12)	1.0940 (10)	0.058 (3)	
H2	0.5251	1.0333	1.0693	0.069*	
C3	0.5700 (14)	1.2794 (13)	1.0380 (11)	0.066 (3)	
H3	0.6527	1.2728	0.9709	0.080*	
C4	0.5312 (14)	1.4300 (14)	1.0756 (12)	0.063 (3)	
C5	0.4075 (13)	1.4426 (12)	1.1802 (12)	0.068 (3)	
H5	0.3828	1.5461	1.2115	0.082*	
C6	0.3232 (12)	1.2979 (11)	1.2355 (10)	0.055 (3)	
H6	0.2402	1.3040	1.3025	0.066*	
C7	0.6108 (16)	1.5928 (14)	1.0174 (12)	0.100 (4)	
H7A	0.7225	1.6490	1.0800	0.150*	
H7B	0.5334	1.6661	1.0256	0.150*	
H7C	0.6267	1.5659	0.9098	0.150*	
C8	0.0860 (11)	0.8191 (9)	0.9787 (8)	0.039 (2)	
C9	0.0942 (10)	0.6546 (8)	0.9560 (9)	0.0405 (19)	
H9	0.0989	0.5959	1.0392	0.049*	
C10	0.0717 (12)	0.9097 (10)	0.8580 (9)	0.049 (2)	
H10	0.0579	1.0193	0.8773	0.058*	
C11	0.0786 (11)	0.8330 (9)	0.7098 (9)	0.050 (2)	
H11	0.0740	0.8936	0.6279	0.060*	
C12	0.0926 (10)	0.6654 (10)	0.6776 (9)	0.040 (2)	
C13	0.0952 (10)	0.5776 (9)	0.8033 (9)	0.041 (2)	
C14	−0.1986 (12)	0.3410 (11)	0.4653 (9)	0.039 (2)	
C15	−0.3270 (10)	0.3498 (10)	0.5546 (9)	0.046 (2)	
H15	−0.3577	0.4514	0.5797	0.055*	
C16	−0.4131 (12)	0.2048 (11)	0.6089 (10)	0.051 (2)	
H16	−0.5036	0.2094	0.6679	0.061*	
C17	−0.3648 (13)	0.0550 (12)	0.5756 (10)	0.054 (3)	
C18	−0.2358 (13)	0.0481 (11)	0.4832 (11)	0.059 (3)	
H18	−0.2063	−0.0540	0.4571	0.071*	
C19	−0.1494 (11)	0.1887 (10)	0.4286 (10)	0.053 (3)	
H19	−0.0600	0.1833	0.3686	0.064*	
C20	−0.4603 (13)	−0.1022 (13)	0.6365 (12)	0.082 (3)	
H20A	−0.5067	−0.1946	0.5501	0.123*	
H20B	−0.5548	−0.0775	0.6878	0.123*	
H20C	−0.3792	−0.1331	0.7097	0.123*	

### Atomic displacement parameters (Å^2^)

**Table:**

	*U*^11^	*U*^22^	*U*^33^	*U*^12^	*U*^13^	*U*^23^
S1	0.0547 (15)	0.0356 (11)	0.0427 (13)	0.0047 (10)	0.0065 (11)	0.0075 (9)
S2	0.0554 (15)	0.0371 (12)	0.0423 (13)	0.0051 (10)	0.0035 (11)	0.0100 (9)
O1	0.097 (6)	0.056 (4)	0.043 (4)	0.009 (4)	0.017 (4)	0.012 (3)
O2	0.071 (5)	0.042 (4)	0.067 (4)	0.017 (3)	−0.001 (4)	0.016 (3)
O3	0.049 (4)	0.043 (3)	0.042 (3)	0.007 (3)	0.007 (3)	0.000 (2)
O4	0.046 (4)	0.048 (3)	0.044 (4)	0.000 (3)	0.011 (3)	0.008 (2)
O5	0.065 (5)	0.039 (4)	0.070 (4)	0.009 (3)	−0.004 (4)	0.009 (3)
O6	0.071 (5)	0.058 (4)	0.038 (4)	−0.002 (3)	0.014 (3)	0.013 (3)
O7	0.127 (5)	0.102 (5)	0.103 (4)	0.080 (4)	0.018 (4)	0.004 (3)
O8	0.169 (6)	0.043 (3)	0.103 (4)	0.029 (3)	0.009 (4)	0.031 (3)
N1	0.099 (5)	0.050 (3)	0.062 (3)	0.029 (3)	−0.016 (3)	0.003 (3)
C1	0.041 (6)	0.034 (5)	0.048 (6)	−0.001 (4)	0.003 (4)	0.006 (4)
C2	0.062 (7)	0.067 (6)	0.050 (5)	0.018 (5)	0.026 (5)	0.009 (4)
C3	0.063 (7)	0.075 (8)	0.055 (6)	0.000 (5)	0.017 (5)	0.016 (5)
C4	0.049 (7)	0.066 (7)	0.066 (7)	−0.004 (5)	−0.007 (5)	0.033 (5)
C5	0.054 (7)	0.043 (6)	0.106 (10)	0.006 (5)	0.005 (7)	0.024 (5)
C6	0.057 (7)	0.040 (5)	0.074 (7)	0.021 (5)	0.013 (5)	0.014 (4)
C7	0.085 (8)	0.093 (9)	0.099 (9)	−0.031 (7)	−0.006 (7)	0.048 (6)
C8	0.039 (5)	0.032 (4)	0.040 (5)	−0.002 (4)	0.006 (4)	0.008 (3)
C9	0.049 (6)	0.030 (4)	0.042 (5)	0.007 (4)	0.003 (4)	0.013 (3)
C10	0.060 (7)	0.028 (5)	0.050 (6)	0.005 (4)	−0.008 (5)	0.005 (4)
C11	0.056 (6)	0.041 (5)	0.046 (6)	−0.004 (4)	−0.003 (4)	0.020 (4)
C12	0.029 (5)	0.040 (5)	0.047 (6)	0.002 (4)	0.001 (4)	0.011 (4)
C13	0.041 (5)	0.033 (5)	0.050 (6)	0.008 (4)	0.006 (4)	0.013 (3)
C14	0.039 (5)	0.043 (5)	0.034 (5)	0.011 (4)	0.003 (4)	0.008 (4)
C15	0.040 (5)	0.036 (5)	0.056 (5)	0.006 (4)	−0.002 (4)	0.002 (3)
C16	0.040 (5)	0.070 (6)	0.047 (5)	0.014 (5)	0.012 (4)	0.018 (4)
C17	0.042 (6)	0.052 (6)	0.062 (6)	−0.004 (5)	0.004 (5)	0.018 (5)
C18	0.058 (7)	0.042 (5)	0.082 (7)	0.016 (5)	0.014 (6)	0.018 (5)
C19	0.048 (6)	0.043 (5)	0.067 (7)	0.004 (4)	0.025 (5)	0.008 (4)
C20	0.064 (7)	0.083 (8)	0.112 (9)	0.011 (6)	0.021 (6)	0.061 (6)

### Geometric parameters (Å, °)

**Table:**

S1—O2	1.396 (6)	C7—H7B	0.9600
S1—O1	1.419 (6)	C7—H7C	0.9600
S1—O3	1.591 (6)	C8—C9	1.360 (9)
S1—C1	1.762 (8)	C8—C10	1.385 (10)
S2—O6	1.422 (5)	C9—C13	1.389 (9)
S2—O5	1.439 (6)	C9—H9	0.9300
S2—O4	1.643 (6)	C10—C11	1.366 (10)
S2—C14	1.752 (9)	C10—H10	0.9300
O3—C8	1.441 (8)	C11—C12	1.403 (10)
O4—C12	1.373 (8)	C11—H11	0.9300
O7—N1	1.189 (7)	C12—C13	1.398 (10)
O8—N1	1.247 (8)	C14—C15	1.355 (10)
N1—C13	1.494 (9)	C14—C19	1.405 (10)
C1—C6	1.357 (10)	C15—C16	1.400 (12)
C1—C2	1.417 (10)	C15—H15	0.9300
C2—C3	1.387 (13)	C16—C17	1.380 (12)
C2—H2	0.9300	C16—H16	0.9300
C3—C4	1.353 (13)	C17—C18	1.374 (11)
C3—H3	0.9300	C17—C20	1.532 (12)
C4—C5	1.419 (13)	C18—C19	1.374 (12)
C4—C7	1.521 (14)	C18—H18	0.9300
C5—C6	1.399 (13)	C19—H19	0.9300
C5—H5	0.9300	C20—H20A	0.9600
C6—H6	0.9300	C20—H20B	0.9600
C7—H7A	0.9600	C20—H20C	0.9600
			
O2—S1—O1	119.0 (4)	C9—C8—O3	118.2 (6)
O2—S1—O3	108.4 (3)	C10—C8—O3	118.1 (7)
O1—S1—O3	102.0 (4)	C8—C9—C13	117.3 (7)
O2—S1—C1	112.4 (4)	C8—C9—H9	121.3
O1—S1—C1	110.6 (4)	C13—C9—H9	121.3
O3—S1—C1	102.5 (4)	C11—C10—C8	117.9 (8)
O6—S2—O5	122.6 (4)	C11—C10—H10	121.0
O6—S2—O4	102.9 (3)	C8—C10—H10	121.0
O5—S2—O4	108.1 (3)	C10—C11—C12	121.8 (7)
O6—S2—C14	110.0 (4)	C10—C11—H11	119.1
O5—S2—C14	108.1 (4)	C12—C11—H11	119.1
O4—S2—C14	103.4 (4)	O4—C12—C13	120.7 (7)
C8—O3—S1	118.2 (5)	O4—C12—C11	122.0 (7)
C12—O4—S2	119.0 (5)	C13—C12—C11	117.3 (7)
O7—N1—O8	125.9 (6)	C9—C13—C12	121.9 (7)
O7—N1—C13	119.0 (7)	C9—C13—N1	116.6 (6)
O8—N1—C13	115.1 (6)	C12—C13—N1	121.2 (7)
C6—C1—C2	122.3 (9)	C15—C14—C19	120.7 (8)
C6—C1—S1	119.7 (7)	C15—C14—S2	120.8 (6)
C2—C1—S1	118.0 (6)	C19—C14—S2	118.4 (7)
C3—C2—C1	116.2 (8)	C14—C15—C16	119.5 (8)
C3—C2—H2	121.9	C14—C15—H15	120.3
C1—C2—H2	121.9	C16—C15—H15	120.3
C4—C3—C2	123.6 (10)	C17—C16—C15	120.4 (8)
C4—C3—H3	118.2	C17—C16—H16	119.8
C2—C3—H3	118.2	C15—C16—H16	119.8
C3—C4—C5	118.8 (10)	C18—C17—C16	119.2 (9)
C3—C4—C7	125.8 (12)	C18—C17—C20	121.1 (9)
C5—C4—C7	115.3 (11)	C16—C17—C20	119.7 (9)
C6—C5—C4	119.3 (9)	C17—C18—C19	121.4 (9)
C6—C5—H5	120.3	C17—C18—H18	119.3
C4—C5—H5	120.3	C19—C18—H18	119.3
C1—C6—C5	119.6 (9)	C18—C19—C14	118.7 (8)
C1—C6—H6	120.2	C18—C19—H19	120.6
C5—C6—H6	120.2	C14—C19—H19	120.6
C4—C7—H7A	109.5	C17—C20—H20A	109.5
C4—C7—H7B	109.5	C17—C20—H20B	109.5
H7A—C7—H7B	109.5	H20A—C20—H20B	109.5
C4—C7—H7C	109.5	C17—C20—H20C	109.5
H7A—C7—H7C	109.5	H20A—C20—H20C	109.5
H7B—C7—H7C	109.5	H20B—C20—H20C	109.5
C9—C8—C10	123.5 (7)		
			
O2—S1—O3—C8	−45.0 (6)	S2—O4—C12—C11	−75.6 (9)
O1—S1—O3—C8	−171.4 (5)	C10—C11—C12—O4	179.9 (7)
C1—S1—O3—C8	74.1 (5)	C10—C11—C12—C13	1.4 (12)
O6—S2—O4—C12	171.7 (5)	C8—C9—C13—C12	1.6 (10)
O5—S2—O4—C12	40.7 (6)	C8—C9—C13—N1	176.1 (8)
C14—S2—O4—C12	−73.8 (6)	O4—C12—C13—C9	177.9 (6)
O2—S1—C1—C6	−160.2 (7)	C11—C12—C13—C9	−3.6 (11)
O1—S1—C1—C6	−24.6 (9)	O4—C12—C13—N1	3.6 (12)
O3—S1—C1—C6	83.5 (7)	C11—C12—C13—N1	−177.8 (7)
O2—S1—C1—C2	18.8 (9)	O7—N1—C13—C9	−132.7 (7)
O1—S1—C1—C2	154.4 (8)	O8—N1—C13—C9	48.5 (9)
O3—S1—C1—C2	−97.4 (8)	O7—N1—C13—C12	41.8 (11)
C6—C1—C2—C3	−4.1 (15)	O8—N1—C13—C12	−136.9 (8)
S1—C1—C2—C3	176.8 (7)	O6—S2—C14—C15	−152.6 (7)
C1—C2—C3—C4	2.3 (16)	O5—S2—C14—C15	−16.4 (9)
C2—C3—C4—C5	1.1 (17)	O4—S2—C14—C15	98.0 (8)
C2—C3—C4—C7	−179.8 (9)	O6—S2—C14—C19	30.7 (8)
C3—C4—C5—C6	−3.0 (16)	O5—S2—C14—C19	166.9 (6)
C7—C4—C5—C6	177.8 (8)	O4—S2—C14—C19	−78.6 (6)
C2—C1—C6—C5	2.4 (14)	C19—C14—C15—C16	−0.9 (13)
S1—C1—C6—C5	−178.6 (8)	S2—C14—C15—C16	−177.5 (6)
C4—C5—C6—C1	1.2 (14)	C14—C15—C16—C17	1.8 (13)
S1—O3—C8—C9	79.1 (8)	C15—C16—C17—C18	−2.6 (14)
S1—O3—C8—C10	−106.1 (8)	C15—C16—C17—C20	179.8 (9)
C10—C8—C9—C13	2.7 (12)	C16—C17—C18—C19	2.6 (15)
O3—C8—C9—C13	177.2 (7)	C20—C17—C18—C19	−179.8 (8)
C9—C8—C10—C11	−4.7 (13)	C17—C18—C19—C14	−1.8 (14)
O3—C8—C10—C11	−179.3 (7)	C15—C14—C19—C18	1.0 (12)
C8—C10—C11—C12	2.5 (12)	S2—C14—C19—C18	177.6 (7)
S2—O4—C12—C13	102.9 (8)		

### Hydrogen-bond geometry (Å, °)

**Table:**

*D*—H···*A*	*D*—H	H···*A*	*D*···*A*	*D*—H···*A*
C5—H5···O2^i^	0.93	2.49	3.405 (11)	168
C9—H9···O6^ii^	0.93	2.46	3.319 (9)	153
C10—H10···O8^i^	0.93	2.47	3.369 (10)	164
C11—H11···O1^iii^	0.93	2.50	3.352 (10)	153
C15—H15···O5	0.93	2.56	2.923 (10)	104
C18—H18···O5^iv^	0.93	2.49	3.409 (11)	168

Symmetry codes: (i) *x*, *y*+1, *z*; (ii) *x*, *y*, *z*+1; (iii) *x*, *y*, *z*−1; (iv) *x*, *y*−1, *z*.
